# Universal health coverage and primary health care: the 30 by 2030 campaign

**DOI:** 10.2471/BLT.19.245670

**Published:** 2020-10-05

**Authors:** Jan De Maeseneer, Donald Li, Bjorg Palsdottir, Bob Mash, Diederik Aarendonk, Anna Stavdal, Shabir Moosa, Peter Decat, Elsie Kiguli-Malwadde, Gorik Ooms, Sara Willems

**Affiliations:** a Department of Public Health and Primary Care, Ghent University, U.Z.-Building 6 K3, Corneel Heymanslaan, 10, B-9000 Ghent, Belgium.; b Hong Kong, Hong Kong Special Administrative Region, China.; c THEnet: Training for Health Equity Network, Brussels, Belgium.; d Faculty of Medicine and Health Sciences, Stellenbosch University, Stellenbosch, South Africa.; e European Forum for Primary Care, Utrecht, Netherlands.; f World Organization of Family Doctors, Bangkok, Thailand.; g Faculty of Health Sciences, University of Witwatersrand, Johannesburg, South Africa.; h Department of Health Workforce Education and Development, African Center for Global Health and Social Transformation, Kampala, Uganda.; i Department of Global Health and Development, London School of Hygiene & Tropical Medicine, London, England.

The World Health Organization (WHO) considers primary health care a cornerstone of universal health coverage (UHC) and describes it as an approach to health and well-being centred on the needs and circumstances of individuals, families and communities. Primary health care should address physical, mental and social health and well-being, and is about providing whole-person care for health needs throughout life, not just treating a set of specific diseases.^1^ We argue that implementing primary health care should focus on broad-based participatory action, including integrated and comprehensive person-centred care, community development and social determinants of health.

## The 30 by 2030 campaign

To prevent most diseases and treat people in a holistic way, community-based primary health-care systems need to be strengthened. Therefore, organizations such as the World Organization of Family Doctors, European Forum for Primary Care, African Forum for Primary Care, Primary Health Care and Family Medicine, as well as The Network: Towards Unity for Health and Training for Health Equity Network are launching the 30 by 2030 campaign in November 2020. These organizations call for international donors to assign 30% of their vertical top-down, disease-oriented budgets to strengthening integrated horizontal community-based primary health-care systems by 2030. The campaign seeks to put into practice Resolution WHA62.12, which urges Member States “to encourage the development, integration and implementation of vertical programmes, including disease-specific programmes, in the context of integrated primary health care.”^2^ This integration should also allow primary health-care services to become more responsive to the health needs of populations.

The campaign has two components: (i) information, analysis and awareness on the impact of vertical programmes on health systems; (ii) encouraging the use of 30% of vertical donor investment to strengthen primary health-care services through coordination, increased human resources and improved infrastructure.

## The first component

The average public expenditure in the WHO Africa Region on non-primary health care (hospitals and specialist care, mostly used by population groups with high incomes) is up to three times higher than the spending on primary health care and prevention.^3^ Only one third of total spending on primary health care comes from governments, and the lower the country’s income, the lower the share. The predominant source of financing for primary health-care services are private and external funds, including out-of-pocket expenses.^3^ Most of these funds are channelled through categorical programmes – that is, programmes that focus on the health problems of specific population subgroups or conditions – with little funding going through integrated primary health-care services, which address health conditions in a holistic manner. In the strategy to achieve UHC, WHO has put forward the target of adding 1% of gross domestic product (GDP) to the budget of primary health care. In low-income countries, adding 1% of GDP to current primary health-care expenditures would increase annual spending in this sector, per capita, from 26 United States dollars (US$) to US$ 33.^4^ Such an increase could help, but new strategies should be explored to further strengthen primary health care.

In 2006, the United States President’s Emergency Plan for AIDS Relief (PEPFAR) provided Zambia with a budget of US$ 150 million targeted for human immunodeficiency virus (HIV), whereas the entire budget of the health ministry was only US$ 136 million. This is an example of unbalanced distribution of health funding, and it continues to occur across sub-Saharan Africa.^5^ In many countries in the African continent, people living with HIV receive free care, food and educational grants for their children, whereas those with other diseases receive poor care and still have to pay out-of-pocket, leading to inequity by disease.

Worldwide, the primary care facilities that address all diseases across the life-cycle, including common illnesses such as diarrhoea, malnutrition and respiratory tract infections, are largely underfunded. In addition, salaries of health-care providers working for donor-funded vertical programmes are often more than double those of equally trained government workers in the fragile public health sector, creating an internal brain drain. Interviews with 796 alumni from a medical school in Uganda found an influence of the PEPFAR projects on career choices of health professionals, with almost half working for an HIV-related nongovernmental organization and only over one third for government. We recommend that donor funds earmarked for HIV be channelled to primary health-care budgets through the 30 by 2030 mechanism, to attract health workers into other disease areas and broaden health-care capacity.^6^

A review on the impact of global health initiatives on recipient country health systems in low- and middle-income countries selected three initiatives that accounted for an estimated two thirds of external funding earmarked for HIV/AIDS: the Global Fund to Fight AIDS, Tuberculosis and Malaria; the World Bank Multi-country AIDS Program; and PEPFAR. The review found the following effects of global health initiatives: distortion of recipient countries’ national health policies, notably through distracting governments from coordinated efforts to strengthen health systems and forcing health systems to adopt vertical, disease-specific projects.^7^

In the second decade of this century, a group of new vertical disease-oriented programmes focusing on noncommunicable diseases were developed by governments and major health actors. Evidence suggests that, the same as for infectious diseases, the best approach to tackle noncommunicable diseases is rather strengthening integrated primary health care. The long-term management of noncommunicable diseases requires much more than the implementation of standardized protocols and access to affordable essential drugs. Such management requires empowering people, reducing barriers to healthy lifestyles and providing care that reflects the values and goals of the individual patient. Evidence of the effectiveness of primary health care in reducing hospital admissions related to noncommunicable diseases is well documented, and multimorbidity among those with such diseases has been shown to be better tackled in primary health care.^8^

Evidence suggests that vertical disease-oriented programmes do not contribute to UHC. For example, in Mozambique, after nearly 15 years of significant foreign aid for health, 65% of the government’s health budget was funded by external donors in 2014. However, the health system coverage has barely changed: in 2014 the health workforce per population ratio was still among the five lowest in the world at 71/10 000, and the number of health facilities per capita was only 1/16 795 in 2015.^9^ The proportion of total governmental health expenditure allocated to health declined from 13.4% (US$ 109 million) in 2006 to 11.9% (US$ 138 million) in 2009 and 7.8% (US$ 481 million) in 2014, moving away from the Abuja target of 15%^9^ (whereby the increase in absolute numbers is due to the increase of GDP and of global government expenditure).

## Second component

The first strategy of this campaign is direct investments to strengthen primary health care. Moreover, the 30 by 2030 campaign advocates for diagonal investments.^10^ Ethiopia offers an example, where the Global Fund, Gavi, the Vaccine Alliance and PEPFAR have collaborated with other donors to increase their financial support, therefore strengthening the primary health-care system. For example, 18.4% of the total Gavi support (US$ 173 million) between 2007 and 2018 was largely focused on the construction of primary health-care facilities, strengthening of the supply chain, laboratory management and training of primary health-care staff.^11^

When major donors launch a call for project proposals focusing on specific health conditions such as HIV, diabetes, mental health conditions, tuberculosis, malaria, or more recently, coronavirus disease 2019 (COVID-19) in low- and middle-income countries, applicants should make clear how they are going to improve primary health-care service delivery and channel 30% of the resources into strengthening such service. This strengthening can be done by financing the cost of integrating the project in the local primary health-care system. Achieving such integration requires contributing to capacity building for primary health care, supporting infrastructure upgrading, strengthening leadership and organization and improving the community’s involvement. However, characteristics of high-quality care such as accessibility, continuity, coordination and person-centredness are also needed. Investment should also be made in assessing the impact of projects on the health system. Those authorities responsible for primary health-care development at project level (national, regional, provincial, district, community) should be involved in implementation. Moreover, together with other agents, the 30 by 2030 campaign will develop action plans to improve the government’s spending and governance efficiency, participatory processes at the community level and monitoring and evaluation processes.

The organizations involved in the campaign, in the spirit of resolution WHA62.12, reiterate the importance to “train and retain adequate numbers of health workers, with appropriate skill mix, including primary health care nurses, midwives, allied health professionals and family physicians, able to work in a multidisciplinary context, in cooperation with community health workers in order to respond effectively to people’s health needs.”^2^ Resources could also be used to improve sustainability through better pay for teams of primary health-care professionals, preventing brain drain.

The COVID-19 pandemic has documented the role primary health-care teams play in preparedness to address the challenges of a new, rapidly spreading disease. Where primary health-care services (community screening and testing, case investigation with support for home-based isolation or quarantine, triage at primary-care facilities) were effective, there was less pressure on hospitals.^12^ Not all countries have been able to respond to the pandemic from an effective primary health-care system, which is why we advocate for the 30 by 2030 campaign (www.30by2030.net). 
